# Myocardial Ischemia: Differentiating between Epicardial Coronary Artery Atherosclerosis, Microvascular Dysfunction and Vasospasm in the Catheterization Laboratory

**DOI:** 10.3390/jcm13144172

**Published:** 2024-07-16

**Authors:** Giovanni Monizzi, Francesca Di Lenarda, Emanuele Gallinoro, Antonio Luca Bartorelli

**Affiliations:** 1 Division of University Cardiology, IRCCS Ospedale Galeazzi-Sant’Ambrogio, 20157 Milan, Italy; francesca.dilenarda@unimi.it (F.D.L.); e.gallinoro@gmail.com (E.G.); antonio.bartorelli@grupposandonato.it (A.L.B.); 2 Department of Biomedical and Clinical Sciences, “Luigi Sacco”, University of Milan, 20122 Milan, Italy

**Keywords:** ischemia, coronary artery disease, microvascular dysfunction, physiology, INOCA

## Abstract

Ischemic heart disease is currently the most common cause of mortality and morbidity worldwide. Although myocardial ischemia is classically determined by epicardial coronary atherosclerosis, up to 40% of patients referred for coronary angiography have no obstructive coronary artery disease (CAD). Ischemia with non-obstructive coronary artery disease (INOCA) has typically been underestimated in the past because, until recently, its prognostic significance was not completely clear. This review aims to highlight differences and patterns in myocardial ischemia caused by epicardial obstructive CAD, coronary microvascular dysfunction (CMD) or vasomotor abnormalities and to elucidate the state of the art in correctly diagnosing these different patterns.

## 1. Introduction

Ischemic heart disease is the most common cause of mortality and morbidity worldwide [1]. Although myocardial ischemia is classically determined by epicardial coronary atherosclerosis, up to 40% of patients referred for coronary angiography have no obstructive coronary artery disease (CAD) [2,3]. Notably, in such cases, the underlying pathology could be coronary microvascular dysfunction (CMD) or vasomotor abnormalities such as macro- or micro-vasospasm. [3] Ischemia with non-obstructive coronary artery disease (INOCA) has typically been underestimated in the past because, until recently, its prognostic significance was not completely clear. Nowadays, we know that this entity is associated with increased cardiovascular risk as well as impaired quality of life and morbidity [4,5,6,7].

Prompt and correct diagnosis of the disease is of pivotal importance and, even if the EURECA registry showed that underutilization of guidelines recommended diagnostic tests, non-invasive imaging examination is the first-line approach to ruling out epicardial disease [8]. Coronary Computed Tomography Angiography (CCTA) in particular, in the last decade, has emerged as a safe and highly effective test to perform in patients at low-to-intermediate risk of CAD [9]. Nevertheless, this exam is not widely available, and a vast proportion of patients undergo invasive coronary angiography. Sometimes, even if the exam is available, practitioners still exhibit strong resistance to adapting to new guidelines [8]. In the context of INOCA, the diagnostic accuracy of coronary angiography is still hampered by several limitations: firstly, low spatial resolution does not allow the visualization of coronary microcirculation; secondly, angiography alone does not assess vascular function and may be insufficient to reveal the real cause of the ischemic events, resulting in an increased risk of the patient undergoing an invasive test [10].

Non-invasive assessment of coronary microvascular dysfunction usually relies on the quantification of coronary flow reserve, and several techniques are available in clinical practice. Position Emitted Tomography (PET) is considered to be the gold standard for non-invasive assessment of myocardial blood flow (MBF), which is measured in mL/min; other promising technologies include cardiac magnetic resonance and perfusion CT, though these have yet to be extensively validated [11]. These techniques have different availability worldwide and a major limitation is that when flow reserve is reduced, CAD needs to be ruled out before a diagnosis of CMD can be established. Furthermore, provocative tests can only be performed during invasive coronary angiography: a diagnosis of vasospasm is carried out by administering intracoronary Acetylcholine during 12-lead ECG monitoring and invasive coronary angiography [12].

A non-conclusive diagnosis can be dangerous for the patient, as it informs the therapeutic management of their condition, with treatments ranging from percutaneous coronary intervention (PCI) for epicardial obstructive disease to different medical therapies specific for each specific endotype of disease. The CorMicA trial demonstrated that a tailored therapy etiologically driven for CMD resulted in better clinical outcomes in terms of quality of life and repeated hospitalization [13].

A comprehensive approach to defining the determinants of myocardial ischemia is now possible thanks to current knowledge and the technologies that are available in the catheterization laboratory [14]. Integrating an invasive physiological assessment of epicardial and microvascular function with a provocative test allows us to easily discriminate epicardial disease from vasospasm and CMD, provides information about specific endotype of the disease, and informs the choice of therapy.

**Figure 1:**
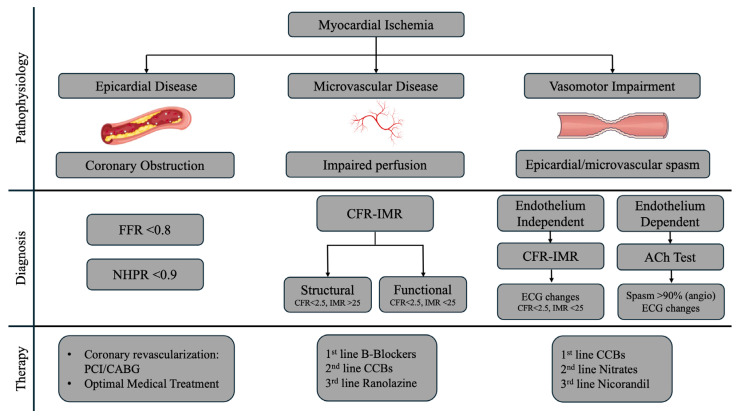
Myocardial ischemia determinants. FFR: fractional flow reserve; NHPR: non-hyperemic pressure ratio; CFR: coronary flow reserve; IMR: index of microvascular resistance: ACh: Acetylcholine, PCI: percutaneous coronary intervention: CABG: coronary artery bypass graft; CCB: calcium-channel blockers.

The aim of this review is to summarize all the possible determinants of myocardial ischemia and offer a pathway (see Appendix A) for clinicians clarifying when and how to assess them comprehensively and easily in the catheterization laboratory (see Figure 1).

## 2. Epicardial Disease

Obstruction of the epicardial coronary is the most acknowledged cause underlying myocardial ischemia. Eye-sight stenosis evaluation during coronary angiography has always played an important role in informing the choice between revascularization and medical therapy. [10] To date, performing physiological assessment of epicardial stenosis has become pivotal both during diagnosis and after revascularization [9].

### 2.1. Diagnosis

*Why*: Moderate and moderate-to-severe lesions have always been treated at the discretion of the operator, who subjectively evaluates their degree of severity. Lesion severity can be determined by discriminating hemodynamically relevant stenosis from the others: moderate-looking disease may prove relevant to the occurrence of ischemia, whereas lesions that initially appeared to be severe may later be deemed not worthy of treatment [15,16].

*When*: This analysis should be applied whenever lesions liable to misquantification are documented and when apparently non-significant disease contrasts with overt symptoms. In addition, evaluation of non-culprit epicardial disease in ACSs may provide guidance for clinicians, helping them to decide whether to extend revascularization to the other lesions (Figure 1).

*What*: The evaluation begins with an assessment of the non-hyperemic pressure ratio (NHPR). The first such ratio to be developed was the instantaneous wave-free ratio (iFR), which consisted of the ratio of distal coronary pressure (Pd) and aortic pressure (Pa) during a specific period of diastole (wave-free period); this is the only non-hyperemic index mentioned in the guidelines and achieves 80% accuracy compared to fractional flow reserve (FFR) [17,18,19,20]. The diastolic hyperemia-free ratio (DFR) consists of an average of five consecutive Pd/Pa measures over the approximated diastolic period; when the same average is calculated at the pressure peak-to-peak midpoint, the diastolic pressure ratio (dFR) is obtained. The resting full-cycle ratio (RFR) evaluates the entire cardiac cycle and not just the diastolic phase. Its threshold is 0.89. The last NHPR is resting Pd/Pa, which is a ratio averaged over the entire cycle, with a cut-off of 0.91.

Fractional flow reserve (FFR) should always be assessed after hyperemia has been induced, as mentioned above. A comprehensive assessment that includes the vasodilatory response to the hyperemic stimulus can be obtained by systematically measuring both non-hyperemic and hyperemic physiology: discordance between NHPR and FFR may occur, and measuring both provides complete information about the functional status of the coronary vessel. RFR is considered to be indicative of ischemia when ≤0.89 and FFR when ≤0.80. To complete the assessment, pressure wire pullback is performed: this technique provides a point-by-point physiological map of the vessel and is used to investigate the pattern of disease (focal, diffuse, or mixed pattern) and to exclude significant drift within the pressure tracer [21]. This last technique can be used interchangeably, and is applicable both at rest and during hyperemia at its steady state, bringing out minor pressure gradients that are otherwise ignored when assessing the latter condition.

A recent review by Scarsini et al. suggested a workflow that would fully investigate the epicardial status and stressed the importance of correlating the results of epicardial assessment whilst also considering CMD, as alterations in microcirculation may be early manifestations of atherosclerosis that can be a precursor to obstructive CAD [14,22].

*How*: Before positioning the pressure-wire downstream of the lesion, equalization is carried out. Hyperemia can be induced by administering intracoronary papaverine (15 mg for the left coronary artery and 10 mg for the right coronary artery), and/or adenosine (200 µg for the left coronary artery and 100 µg for the right coronary artery), and/or contrast medium: if pharmacological hyperemia is to be avoided, the contrast fractional flow reserve (cFFR) is an efficient surrogate of FFR, taking advantage of the vasodilatory properties of contrast medium [23].

Furthermore, intravenous adenosine is a valid alternative (140 mg/kg/min) and should be preferred over intracoronary adenosine, as it allows more reproducible measurements and longitudinal pullback in case of a positive value.

Before inducing hyperemia, it is advisable to administer nitrates to reverse any eventual spasms.

*Procedural safety.* Before administering papaverine, it is mandatory to fully flush the catheter with saline in order to remove any remaining contrast medium residue: this could trigger ventricular tachycardia/fibrillation if it comes in contact with papaverine. Prior to adenosine administration, it is advisable to have atropine already at one’s disposal, in case of atrioventricular block.

*Possible Scenarios and related therapy.* Negative FFR and RFR with no pressure drop at pullback result in an absence of significant epicardial pathology. “Focal” hemodynamically significant lesions are identified when FFR and RFR increase by more than 0.05 and 0.03, respectively, upstream of the stenosis, and when a sharp pressure drop is observed at pullback in a short vessel segment (≤20 mm). When two or more of these are present, separated by a non-diseased vessel segment (>20 mm), the condition is referred to as “serial lesions”. A progressive decrease in the pressure gradient at a pullback in the absence of a clear pressure drop and the presence of positive FFR is defined as obstructive “diffuse disease”. When both focal and diffuse lesions are present, mixed patterns are defined and classification depends on the predominant feature: type I is mainly focal, type II is mainly diffused, and type III has a balanced distribution. Coronary angioplasty is the preferred therapy when focal lesions are documented and medical therapy is usually recommended if no significant pressure drops are observed. Diffuse disease is associated with suboptimal patient-reported outcomes after PCI [24]. In case of discrepancy, abnormal RFR tends to be associated with predominantly diffuse disease, whereas abnormal FFR tends to be associated with a predominantly focal pattern of disease [21].

### 2.2. Post-PCI Assessment

*Why:* Optimal angiographic results following PCI do not always correspond to fully successful flow restoration [25]. The aim of post-revascularization FFR is to achieve a higher value than 0.90 to ensure an adequate outcome [25].

*When:* Post-PCI physiology assessment should always be performed in order to intercept complications such as residual stenosis, coronary spasm, or stent-related complications.

*How and What:* The materials and methods are mentioned above; thresholds vary between right coronary artery (RC) and left circumflex (LCx), both of which refer to 0.90 and the left anterior descending artery (LAD), which has 0.86 as a cut-off; any result above this threshold is considered acceptable.

The role of IMR measurement in post-PCI has proved to be strongly predictive of long-term outcomes in STEMI patients [26]. Adverse events correlated with an IMR > 40 include death alone and hospitalization or death following heart failure [27].

Further studies need to be conducted to validate a value of post-PCI IMR in non-STEMI patients.

**Figure 2:**
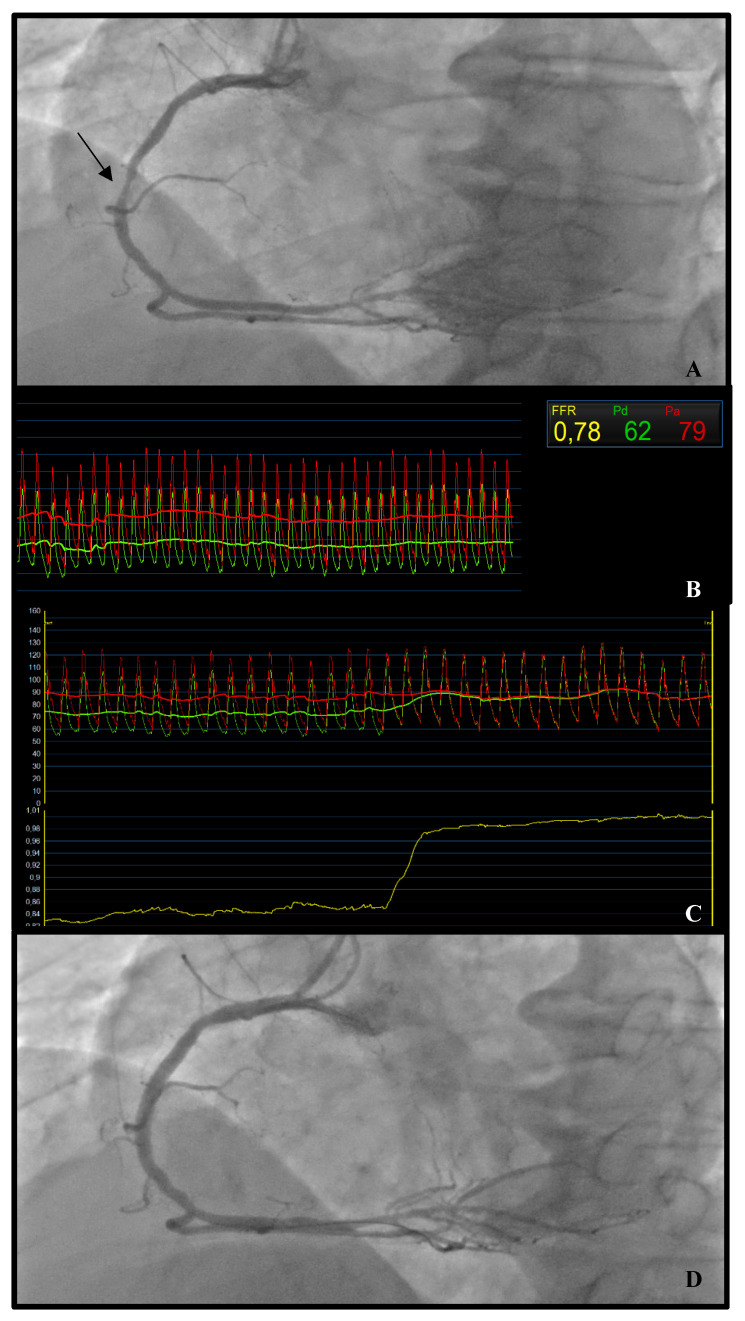
***Epicardial Disease.*** Scan of a 72-year-old man presenting with acute chest pain and anterior STEMI at ECG. Basal angiography documented LAD occlusion and visually moderate RCA stenosis (**A**, black arrow). Functional evaluation was performed on the latter, non-culprit lesion. FFR came out positive (**B**) and a significant pressure drop was documented at pullback (**C**). In presence of a focal, significant lesion, PCI was therefore performed, resulting in an optimal angiographic result (**D**).

An example of epicardial disease is shown in Figure 2.

## 3. Microvascular Disease

*Why:* A coronary angiogram of patients presenting with angina and those presenting with infarction may reveal an absence of obstructive coronary artery disease (CAD), defining two phenotypes called ANOCA and INOCA, respectively. Coronary microvascular dysfunction (CMD) underlies the two conditions and its diagnosis guides following medical therapy, which makes the test pivotal for adequate patient management (Figure 2).

*When:* Microcirculation should be examined in the presence of ANOCA and INOCA patients and whenever inducible myocardial ischemia detected with non-invasive tests finds no epicardial significant lesions on a coronary angiogram [28]. Furthermore, investigation of microvascular resistance may be of great importance in the presence of epicardial disease: both pre-existing dysfunction and the resulting PCI impairment are linked with relevant myocardial injury [29]. Finally, ongoing studies are investigating the role of potential intracoronary treatments to revert microvascular damage following PCI performed in Acute Coronary Syndromes.

*How:* Functional assessment of non-epicardial myocardial vascularization is commonly performed via bolus thermodilution. In addition to materials required for the analysis mentioned above, 3 mL syringes are required. Specifically, the pressure wire is positioned distally in LAD (or in the coronary artery which serves the largest amount of myocardium) and to follow, three injections of room-temperature saline are administered through a guiding catheter at rest and after the induction of hyperemia. The distal intracoronary pressure and mean transit time (Tmn, in sec; that is, the mean transit time the indicator takes to travel from the injection site to the distal pressure–temperature sensor of the wire) are measured.

More recently, evaluation of coronary microvascular dysfunction has been investigated via continuous thermodilution, which was performed injecting intracoronary room-temperature saline in a monorail infusion catheter.

*What:* The indexes measured by the software after bolus thermodilution is performed are coronary flow reserve (CFR_bolus_), IMR, and resistance reserve ratio (RRR). CFR is the ratio between Tmn at rest and Tmn in hyperemia, and it is pathological when lower than 2.0. On the other hand, IMR, an index of microcirculatory resistance, is obtained by multiplying distal pressure by Tmn during hyperemia; a diagnostic threshold of microvascular dysfunction has been identified for IMR above 25. RRR is not commonly used; it expresses the ratio between basal and hyperemic microvascular resistance, and it is correlated to a patient-oriented composite outcome when lower than 3.5 [30].

Continuous thermodilution allows for the quantification of absolute coronary flow (Q) in mL/min, both at rest and during hyperemia and microvascular resistance (R*_μ_*) in WU. Subsequently, CFR_cont_ and microvascular resistance reserve (MRR, that is, the ratio between CFR and FFR corrected for the driving pressures) can be derived. Despite the higher reproducibility of continuous termodilution-derived indices, the widespread adoption of this technique is still limited by the availability and cost of the RayFlow catheter [31]. The clinical impact and routinary application are to be investigated in future studies.

*Possible scenarios and related therapy.* Three main phenotypes of microvascular dysfunction may be detected: structural, functional, and compensated CMD. The first consists of a histological alteration of microcirculation, deriving from prearteriolar, arteriolar, and capillary remodeling (luminal narrowing, perivascular fibrosis, and capillary rarefaction) which results in increased microvascular resistance and inadequacy of myocardial perfusion following increase in oxygen demand [32]. A diagnosis of Structural CMD requires CFR < 2.0 and IMR > 25; histologically, these patients may represent a group with arteriolar obliteration, microvascular obstruction, and/or capillary rarefaction. This phenotype correlates with a higher prevalence of exercise-related hypertension and impaired systemic vasodilatation: the increase in coronary flow may be inhibited, resulting in reduced hyperemic flow due to high minimal microvascular resistance [33].

In the presence of normal IMR (<25) but pathological CFR (<2.0), pathophysiological alterations consist in impaired vasodilatation in the presence of unaltered microvascular resistance; this condition is defined as Functional CMD. This group of patients has increased baseline coronary blood flow due to reduced microvascular resistance at rest, as a response to either impaired coronary autoregulation or increased myocardial oxygen demand at rest, mediated by increased activity of the NOS pathway [33].

The last possible scenario corresponds to finding altered IMR in presence of normal CFR, which has yet to be defined as a clinical condition. Current hypotheses suggest this may reflect early compensated damage. Initial data suggest a lack of prognostic impact on isolated pathological IMR, considering these patients comparable to patients in whom both CFR and IMR result normal [34].

**Figure 3:**
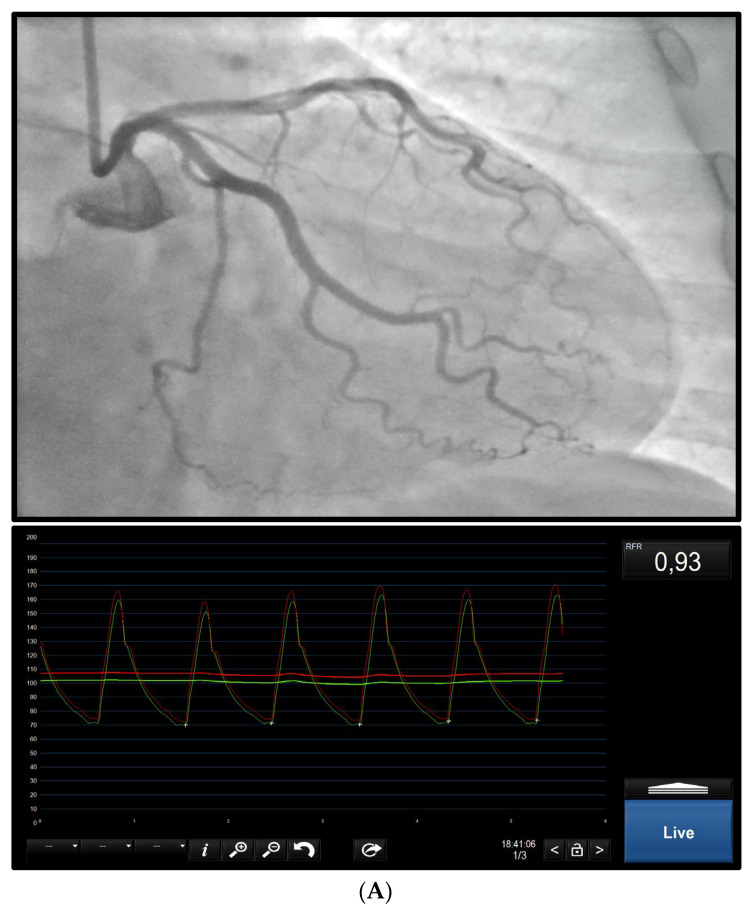

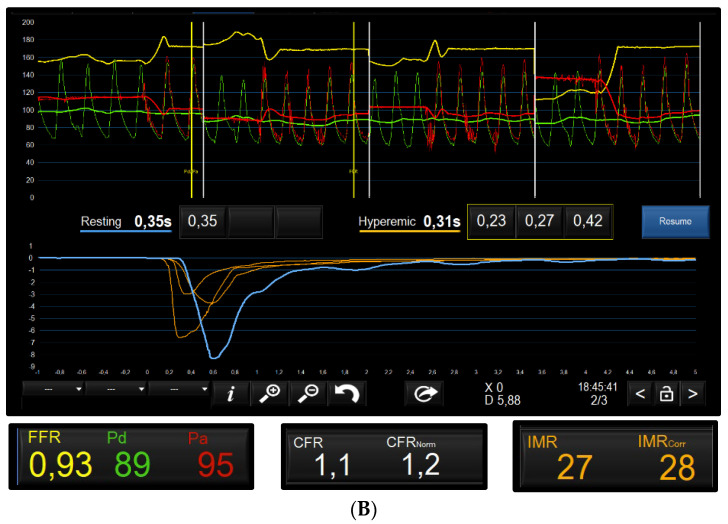
***Microvascular Disease.*** The results of a 65-year-old woman presenting with exertional chest discomfort. Angiography revealed no epicardial stenosis (**A**). Functional evaluation was performed (**B**) and normal RFR and FFR were documented. CFR was found to be altered (<2.0), as was as IMR (>25). Microvascular structural disease was diagnosed and medical therapy was optimized, introducing lipid-lowering therapy, ACE inhibitors, and beta-blockers.

Treatment involves lifestyle changes and medical therapy. The former consist of interventions targeted to reduce major cardiovascular risk factors via regular physical activity, smoking cessation, modification of dietary habits, and weight loss. The latter involves introducing medical therapy to improve afterload reduction and vascular remodeling, on top of lowering blood cholesterol and hypertension: beta-blockers (BB), ACE inhibitors and statins. Calcium-channel blockers are to be reserved for BB-intolerant patients. Other effective treatments include anti-anginous therapies such as nicorandil and ranolazine; ivabradine, trimetazidine, and tricyclic antidepressants can also be considered. As far as functional CMD is concerned, targeted therapy should aim to raise the basic myocardial metabolism, which is to be investigated in future studies [34,35]. An example of microvascular disease is shown in Figure 3.

## 4. Vasomotor Impairment

*Why:* Up to one-third of patients with ischemia and non-obstructive coronary disease present with vasomotion disorders related to both epicardial microvascular circulation [36].

Vasomotor impairment is characterized by enhanced reactivity of vascular smooth muscle, resulting in spasm or impaired vasodilation of the microvascular and/or epicardial compartments [36].

It can be endothelium-independent, reflecting the coronary flow reserve’s inability to adapt to the vasodilatory stimuli (in presence of a reduced CFR < 2 with a normal IMR < 25 and normal FFR > 0.8), or endothelium-dependent with a paradoxical response at Acetylcholine (Ach) administration (Ach usually stimulates endothelium NO production, but if dysfunctional, it binds to muscular smooth cells and determines muscular contraction and spasm) [11].

These conditions are a frequent underdiagnosed cause of ischemia, and are particularly prevalent in women; they represent a threat to the quality of life. The WISE study showed a two-fold increase in recurrent angina hospitalizations in the non-obstructive group at one year follow-up compared to hospitalizations of women with obstructive coronary artery disease [37].

A substudy considering 5-year follow-up demonstrated that in patients with INOCA, abnormal coronary endothelium-dependent vasoreactivity was associated with angina hospitalization, giving us an idea of the importance of this diagnosis [38].

Most of the time, these conditions may coexist with obstructive CAD or CMD [36].

*When:* For the features mentioned above and the epidemiology of the disease, vasomotor function should always be tested in any patient presenting with angina or documented ischemia and no evidence of obstructive CAD or CMD. (Figure 3) Due to the frequent coexistence of these three diseases, vasomotor tests should be always considered to complete the panel of myocardial ischemia tests, making it possible to verify the presence of vasomotor impairment and distinguish between endothelial-dependent and -independent endotypes to correctly target the therapies.

*How, Where and What*: The assessment of endothelium-independent function involves the use of thermodilution-based indexes, namely the index of IMR and CFR, as described in the microvascular section [39].

Endothelium-dependent vasomotor function testing requires 12-lead ECG and symptom monitoring during Ach intracoronary infusion at incremental dosage, and this is usually performed in LAD. This test is usually not carried out in RCA, as Ach may cause atrioventricular blocks. Infusion of Ach consists of three sequential manual slow-rate infusions of incremental doses of Acetylcholine (20–50–100 μg) over a period of two minutes via the guiding catheter. At the end of every infusion, 12-lead ECG must be analyzed and the catheter needs to be slowly flushed with saline before the contrast check to avoid rapid leftover ACh injection. If the spasm is documented, intracoronary nitroglycerin injection (100–200 μg) is performed to alleviate symptoms, as it reverts the effect. Epicardial spasm is defined as the presence of chest pain, ischemic ECG changes, and ≥90% epicardial coronary artery vasoconstriction compared to angiography after nitroglycerin administration [39]. Microvascular spasm is defined as the presence of typical chest pain, ischemic ECG changes, and no evidence of epicardial spasm [11]. Some authors suggest that this test can be conducted by keeping the pressure wire in the vessel to measure NHFR to monitor epicardial spasm (a significant reduction from baseline value) at the end of each ACh infusion to avoid angiography after each step [40] Moreover, it makes it possible to monitor microvascular spasm by measuring the mean transit time (surrogate of IMR) after every step.

*Possible scenarios and therapy*. The exclusion of vasomotor impairment can be carried out only in the presence of a normal CFR (adequate response to hyperemia) with no macrovascular or epicardial spasm after ACh test. If no obstructive CAD or CMD is documented, we can state that a patient is suffering from non-cardiac symptoms. Documentation of ischemia to a previously performed non-invasive test may therefore be a false-positive test (in the absence of myocardial bridging) or a global ischemic discrepancy due to other causes (e.g., anemia, toxicity).

When macrovascular spasm is documented, beta-blockers are absolutely contraindicated and calcium-channels blockers are strongly recommended. In case of microvascular impairment, nitrates or Nebivolol are recommended. Ranolazine and ivabradine are considered a second line of treatment if the first treatment is not sufficient [41].

**Figure 4:**
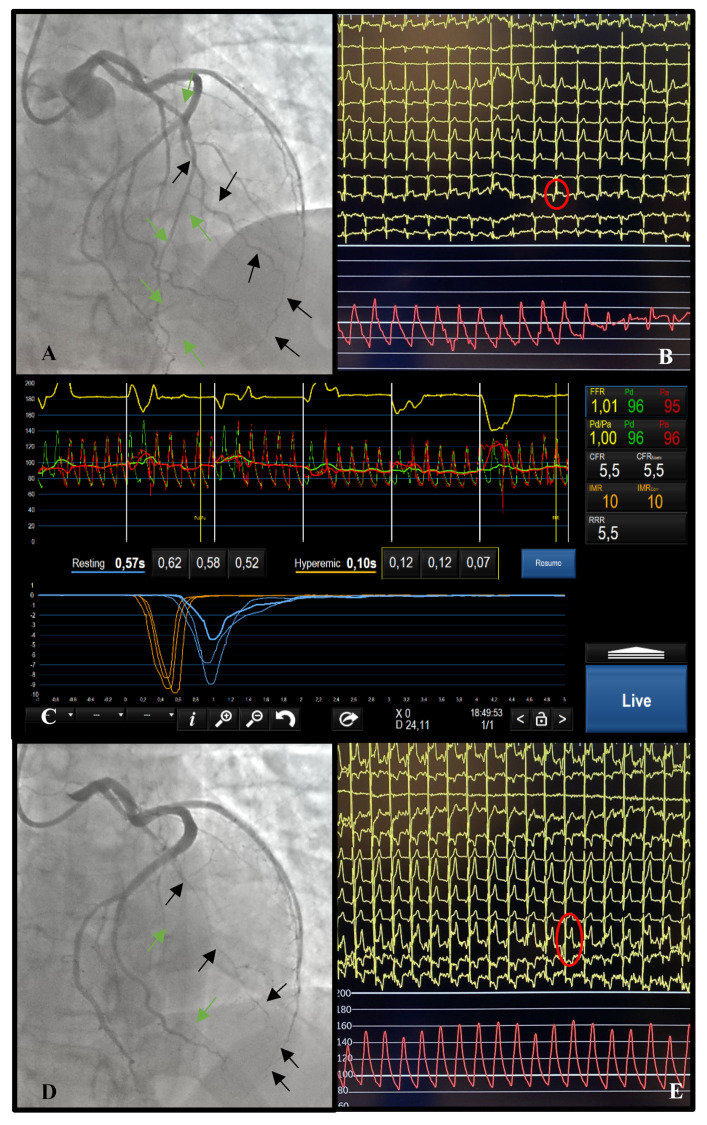
***Vasomotor impairment.*** Results of a 45-year-old woman presenting with acute chest pain and STEMI at ECG. Basal angiography (**A**) and ECG (**B**) performed during symptom regression documented no epicardial stenosis and no electrocardiographic alterations. Black arrows mark a large second marginal branch; green arrows mark LAD. Full physiology documented negative FFR, CFR and IMR (**C**). Acetylcholine test was subsequently performed: arrows point out how both LAD and MO2 undergo important vasospasm (**D**); contextual ST-elevation is documented at 12-lead ECG monitoring (**E**) and intense chest discomfort was reported by the patient. Calcium-channel blockers were introduced.

*Procedural safety.* For CFR, refer to the “Epicardial” section. For the Ach test, nitrates must be readily administered in case of spasm, which must be reverted promptly, preventing persistent symptoms and hemodynamic instability. If blocks (both atrioventricular and sinus blocks) occur, atropine should be immediately administered intravenously to promptly restore sinus rhythm. It is rare to encounter major blocks following Ach administration in the left coronary artery. If vasomotor impairment is present, it usually affects both coronaries, and for this reason, conducting this test in RCA may be useless and risky. The presence of permanent blocks that require a temporary pacemaker is anecdotical, so we would discourage clinicians from making such preparations upfront. An example of vasomotor impairment is shown in Figure 4.

## 5. Future Perspectives

The physiological assessment of coronary circulation is currently of extreme importance in the diagnostic workup of ischemic heart disease, allowing us to correctly differentiate between epicardial disease, CMD, and vasomotor impairment.

The extensive utilization of these tools and techniques will, in future, allow us to become better acquainted with the presence of this kind of disease. This could lead to the development of more precise tools for differentiation and would help us to seek and provide therapies more specifically targeted toward each type of disease.

### Limitation

Comprehensive assessment of coronary physiology is not possible at all locations, as not all catheterization laboratories possess the equipment needed to perform such an analysis. For this reason, the use of this technique is limited and this analysis is more expensive than an angiography alone. Moreover, the exam could be time-consuming and might lead to a reduction in the number of procedures performed per day if not adequately organized in a structured program. Nevertheless, the use of this technique will hopefully become broader and more easy to use in the next few years if more evidences about its cost-effectiveness ratio can be demonstrated and as clinicians gain more experience with it.

Another limitation is that there is a huge variability between patients and intra-patient between different coronary arteries, with significance levels validated by studies mainly conducted on the left anterior descending artery [42]. Much more experience and more studies are needed to determine whether different cutoffs should be set for different arteries or particular scenarios.

## 6. Conclusions

Assessment of CMD is becoming a daily practice and is allowing us to better understand the physiopathology of ischemic heart disease. Moreover, correlation between CMD and poorer prognosis than epicardial coronary disease has been established, and this requires research and clinical efforts to be carried out in order to improve therapeutic strategies.

Percutaneous coronary intervention is becoming more and more targeted and adequate planning and stenosis evaluation is becoming possible thanks to invasive functional assessment, improving patients’ prognosis and limiting adverse outcomes.

Comprehensive physiological coronary assessment is therefore mandatory in symptomatic patients and non-obstructive coronary artery disease and, to better assess epicardial lesions, ongoing studies are further investigating its clinical impact and prognostic relevance.

This review represents a practical guide to help the community of cardiologists dealing with ischemic heart disease. The broader introduction of these tools in daily practice will provide the basis for a better and more extensive comprehension of the most common cause of mortality and morbidity worldwide.

## Figures and Tables

**Figure A1:**
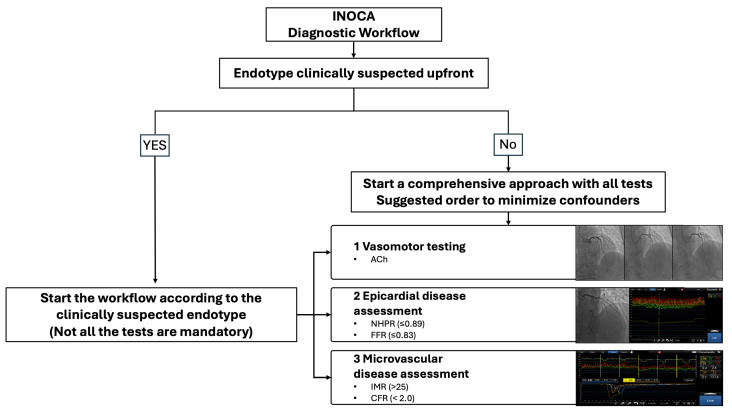
INOCA Diagnostic workflow. A proposed workflow chronologically based on the clinical suspicion.

## Data Availability

No new data were created or analyzed in this study.
